# Modern Medico-Psychology and Psychiatry

**Published:** 1895-06-01

**Authors:** T. S. Clouston

**Affiliations:** Lecturer on Mental Diseases, Edinburgh University, and Physician Superintendent Royal Asylum, Morningside


### Modern Medico-Psychology and Psychiatry.

By T. S. Clouston, M.D., Lecturer on Mental Diseases, Edinburgh University, and Physician Superintendent Royal Asylum, Morningside.

### THE NERVOUS DEFECTS AND DISEASES OF DEVELOPMENT.

Before describing shortly some of the ordinary forms of the clinical insanities, I shall first glance at a very complicated but most important subject, without some comprehension of which one cannot rightly understand some of them at all. To look at mental defects and disorders, apart from the other non-mental nervous diseases with which they are so often associated, hereditarily and personally, would be to take up but a partial and incorrect view of them. To understand in any useful way the significance of the maniacal attacks to which certain youths of both sexes are subject at puberty, or during adolescence, we must look at the brain qualities which such persons, and their fathers and mothers, brothers, sisters, and cousins, have possessed from their birth and during their development, and the nervous diseases they all have been subject to. We must ask also some questions as to what the growth and development of the brain means. The process through which an organ that at birth only exhibits a few simple automatic acts, gradually becomes the vehicle of the most harmonious and complicated movements, of the finest and most delicate sensations, and above all of the highest thought and will, is one of intensest interest to the physiologist, psychologist, and the physician. This period of twenty-five years—for the brain does not reach full development in average persons till that age—may be divided into two, one of growth in bulk and development of function combined, and the other of development of function only. If we take its mere gross bulk and weight, the brain has reached its maximum within a few ounces at seven years of age, and its full size at seventeen. From birth to seventeen would therefore be the period of growth, and from seventeen to twenty-five that of development of function only. During the first period the cells of the grey matter increase in numbers enormously, and the connecting fibres forming the white matter also multiply and form new connections between different groups and levels of cells. During the second period no doubt the cells develop in quality, in intensity, and in delicate reactiveness to all stimuli from within and from without; and above all, new groupings are formed, each with a definite purpose, and still more connections are established between those groupings. The nerve currents become gradually, and by repetition, more definite in their course; the channels of communication between the ever-increasing foci of impressions in the brain cortex from the incoming nerves of the body, and especially of the special senses, become more effective. The brain correlatives of reflection, experience, acts of attention, comparison, self-control, and states of emotion, are all increasing and solidifying. The incomparable mechanism of the human brain cortex is every day becoming more perfect through higher organisation of its matter, and a more constant use of its parts. As a machine, the brain is acquiring by this repeated use more differentiation and more solidarity. The great function of the reproduction of the species arises towards the end of growth, with all its cataclysmal effects on body and mind. Altruism, poetry, morality, all become real forces of life in its higher aspects. It is as if a great metropolis were being supplied with a telegraphic system. First the batteries have to be made, then the wires of communication have to be laid and connected with the batteries, then the centres and the circuits have to be formed, simple at first, then more and more complicated. The operators have to be trained, each working slowly and with difficulty at first, and gradually becoming more expert and automatic. The great defect of this analogy is that one is a system which is by comparison simplicity itself, while the other is by far the most complex mechanism and organisation in nature. The human brain is in structure and function the highest evolution yet reached, and it seems the end and aim of all other evolutions. It contains within itself from the first the stimulus of all its future growth, and the potentialities of all its future powers and capacities. But it also contains, having received them by heredity, the seeds of most of the defects and diseases to which it is subject. These defects first come out during development. They are multiform, and affect every function. Hence we cannot take one of them, such as insanity, and study it by itself. Nutritional diseases, motor diseases, and sensory diseases must be taken together. Evil hereditary tendencies show themselves sometimes before birth, for from nutritional incapacities we see children born with harelip, cleft-palate, idiotic heads, or no heads at all, deformed palates, and atrophied limbs. No doubt, the stronger the evil heredity the earlier it shows itself. There are distinct kinds of such bad heredity, but all are related. Through some of them such nutritional defects and formative arrests as I have referred to take place; through others the motor centres are weakened, and we have in consequence paralytic and convulsive affections; through others we have the mental centres weakened, and we have thereby idiocy and the developmental insanities caused.

During the period of largest growth in bulk, from birth up to seven years, the brain is most subject to defects and disorders which cause the convulsions of teething, infantile paralysis, child epilepsy, stammering, backward speech, squints, some kinds of mental imbecility, great liability to febrile delirium at comparatively low temperatures, tubercular meningitis, rickets, hydrocephalus, child melancholy, and child mania (very rare), deaf-dumbness, &c., &c. During the subsequent and higher development of the brain, from seven to puberty, St. Vitus' dance, asthma, and somnambulism are most apt to arise. From the great era of puberty onwards through the adolescent period, that is, from thirteen to twenty-five, a whole crop of nervous disorders and defects is met with, such as epilepsy, hysteria, megrim, dwarfishness, ugliness, dipsomania, moral perversions, perverted and uncontrollable impulses, consumption, acute rheumatism, and last of all in time adolescent insanity*. These all represent developmental pitfalls and weaknesses in the course of the onward progress of the brain till it arrives at full maturity. Every one of all these, and many more that I have not named, are related to each other, and cannot be properly understood or treated each by itself. To consider one without reference to the others or to their common source in a defective brain, would be entirely unphysiological. Some of them result from partial arrests, *i.e.*, stoppage of development in one brain function, while the other functions go on maturing normally. One thing they all have in common, a bad nervous heredity. Some of them can be met by suitable conditioning and by curative measures, others are entirely beyond any such help, and are nature's seals to denote a stoppage and extinction of a bad stock.

* This subject is more fully treated in the author's "Neuroses of Development."

